# Selective Formation of Intramolecular Hydrogen-Bonding Palladium(II) Complexes with Nucleosides Using Unsymmetrical Tridentate Ligands

**DOI:** 10.3390/molecules27072098

**Published:** 2022-03-24

**Authors:** Ryoji Mitsuhashi, Yuya Imai, Takayoshi Suzuki, Yoshihito Hayashi

**Affiliations:** 1 Institute of Liberal Arts and Science, Kanazawa University, Kakuma, Kanazawa 920-1192, Ishikawa, Japan; 2 Department of Chemistry, Kanazawa University, Kakuma, Kanazawa 920-1192, Ishikawa, Japan; helibnnaalclkvcu@stu.kanazawa-u.ac.jp (Y.I.); hayashi@se.kanazawa-u.ac.jp (Y.H.); 3 Research Institute for Interdisciplinary Science, Okayama University, 3-1-1 Tsushima-naka, Okayama 700-8530, Japan; suzuki@okayama-u.ac.jp

**Keywords:** palladium(II) complex, hydrogen-bonding interactions, crystal structure, nucleoside

## Abstract

Three palladium(II) complexes with amino-amidato-phenolato-type tridentate ligands were synthesized and characterized by ^1^H NMR spectroscopy and X-ray crystallography. The strategic arrangement of a hydrogen-bond donor and acceptor adjacent to the substitution site of the Pd^II^ complex allowed the selective coordination of nucleosides. Among two pyrimidine-nucleosides, cytidine and 5-methyluridine, cytidine was successfully coordinated to the Pd^II^ complex while 5-methyluridne was not. On the other hand, both purine-nucleosides, adenosine and guanosine, were coordinated to the Pd^II^ complex. As purines have several coordination sites, adenosine afforded three kinds of coordination isomers expected from the three different donors. However, guanosine afforded a sole product according to the ligand design such that the formation of double intramolecular hydrogen-bond strongly induced the specific coordination by *N1*-position of guanine moiety. Furthermore, the preference of the nucleosides was evaluated by scrambling reactions. It was found that the preference of guanosine is nearly twice as high as adenosine and cytidine, owing to the three-point interaction of a coordination bond and two hydrogen bonds. These results show that the combination of a coordination and hydrogen bonds, which is reminiscent of the Watson–Crick base pairing, is an effective tool for the precise recognition of nucleosides.

## 1. Introduction

Hydrogen-bonding interactions have attracted much attention in the past and present as they are involved in many chemical and biological systems. Hydrogen bonds exhibit a directionality and reversibility upon bond formation. Although the bond energy is much smaller than that of a covalent bond, such unique properties enable construction of supramolecular structures of a molecule to control the chemical and physical properties [1,2,3,4,5,6,7,8]. Furthermore, it is also possible to precisely recognize a molecule by tuning the relative position of hydrogen-bond donors and acceptors in a molecule as represented by the formation of the base pair in DNA [9,10].

**Figure 1:**
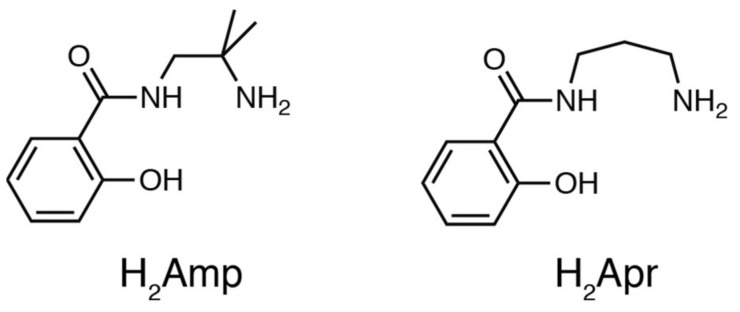
Chemical structures of ligand precursors H_2_Amp and H_2_Apr.

Coordination of biomolecules such as nucleobases to a transition metal ion often exhibits cytotoxic properties [11,12,13,14]. For example, a well-known mononuclear Pt^II^ complex, cisplatin *cis*-[PtCl_2_(NH_3_)_2_], inhibits the replication of DNA by selective coordination by two continuous guanine residues of DNA. On the other hand, we previously reported synthesis and crystal structures of cobalt and manganese complexes with amino-amidato-phenolato-type tridentate ligands (Figure 1) [15,16]. In combination with a Pd^II^ ion, for which square planar geometry is expected, the amino-amidato-phenolato ligand affords a vacant coordination site for the recognition of nucleosides by arranging a hydrogen-bond donor and acceptor to accommodate a specific nucleoside. For this Pd^II^ complex fragment, selective coordination of a nucleobase is expected, owing to a labile coordination bond in Pd^II^ complex and the arrangement of hydrogen-bonding sites. In this study, we synthesized Pd^II^ complexes with two amino-amidato-phenolato-type ligands and evaluated the effects of hydrogen-bonding interaction on the selectivity of nucleosides upon coordination to the Pd^II^ complex fragment. For such tridentate ligands, it is expected to form a Pd–N coordination and two intramolecular hydrogen bonds with guanine and cytosine moieties. We demonstrated that this three-point interaction enables the precise recognition of nucleobases.

## 2. Results and Discussion

### 2.1. Preparation

#### Preparation of Pd^II^ Complexes

**Figure 2:**
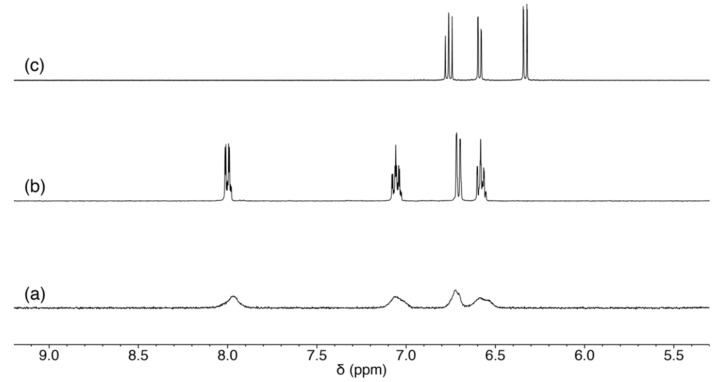
^1^H NMR spectra of (**a**) **1**, (**b**) **1′** in CD_3_OD, and (**c**) **2** in CD_3_CN.

The ligand precursors H_2_Amp, H_2_Apr, and Pd^II^ starting material were prepared according to the previously reported procedures [15,17]. The Pd^II^ complex was synthesized by a reaction of [PdCl_2_(CH_3_CN)_2_] and H_2_Amp followed by an addition of triethylamine and N(CH_3_)_4_OH·5H_2_O as a base in methanol. The product was crystallized by diffusing diethyl ether vapor into the methanol solution to afford yellow crystals of N(CH_3_)_4_[Pd(Amp)Cl]·H_2_O (**1**). To accept the coordination by nucleosides, the presence of chloride ligand is not suitable because of its coordination ability. When the ^1^H NMR spectrum of this product was measured in CD_3_OD, it exhibited rather broad resonances (Figure 2a). This implies that the complex [Pd(Amp)Cl]^−^ undergoes a rapid exchange reaction of Cl^−^ ligand with a solvent methanol molecule. Thus, we exploited this reaction to isolate a chloride-free complex, [Pd(Amp)(CH_3_CN)]·CH_3_CN (**1′**), by using KO*^t^*Bu instead of N(CH_3_)_4_OH·5H_2_O. In this reaction, potassium salt of **1** was not obtained; instead, **1′** was selectively produced due to the elimination of KCl from the reaction mixture. **1′** is more suitable for the substitution reaction with a nucleoside because acetonitrile is a better leaving group.

An analogous Pd^II^ complex with Apr^2−^ was also prepared by a similar procedure with **1′**, and [Pd(Apr)(CH_3_CN)] (**2**) was obtained. Although the ligand structure of Apr^2−^ is analogous to Amp^2−^, **2** exhibited a different coordination mode of the tridentate ligands. The ^1^H NMR spectrum in CD_3_CN only showed three aryl-H resonances, which indicates that one of the protons on the aromatic group was removed by the base (Figure 2c). The elimination of the proton induced the formation of a Pd–C bond, and the resulting direct coordination of the ring carbon makes the acetonitrile coordination site rather hydrophobic. The direct coordination of the aromatic ring was also supported by the significant up-field shift of aryl-H resonances. The formation of **2** has parallels with orthometalation reaction in organometallic chemistry.

### 2.2. Crystal Strucre of Pd^II^ Complexes

#### 2.2.1. Crystal Structures of **1** and **1′**

**Figure 3:**
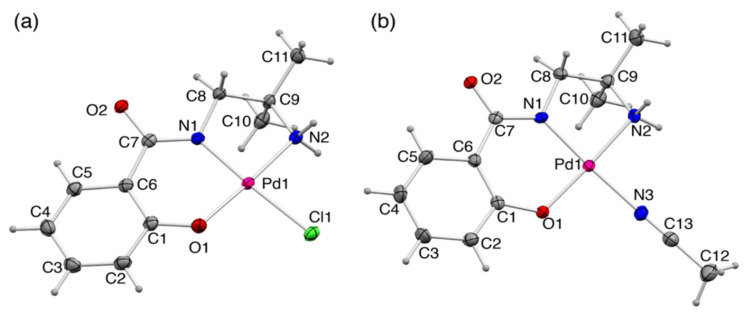
Perspective views of (**a**) [Pd(Amp)Cl]^−^ in **1**, and (**b**) [Pd(Amp)(CH_3_CN)] in **1′** (50% probability levels).

Single-crystals of **1** and **1′** were prepared by diffusing diethyl ether vapor to the reaction solution or slow evaporation of the solution, respectively. The crystallographic information is summarized in Table 1. In both crystals, the Pd^II^ ion takes general square planar coordination geometry (Figure 3). In both **1** and **1′**, Amp^2−^ ligand coordinates to a Pd^II^ center with a phenolato-*O*, amidato-*N*, and amino-*N* atoms as a tridentate ligand. In **1**, the remaining coordination site is occupied by a Cl^−^ ion to afford an anionic Pd^II^ complex, [Pd(Amp)Cl]^−^. In **1′**, on the other hand, the Pd^II^ center is coordinated by an acetonitrile molecule instead of Cl^−^ ligand, although the reaction solvent was acetonitrile in both cases. This is because the Cl^−^ ion was removed from the system during the reaction due to low solubility of KCl. The bonding parameters around the Pd^II^ ion are summarized in Table 2. The bond lengths around the Pd^II^ center are nearly identical between **1** and **1′** except those of the ancillary ligand Cl^−^ and CH_3_CN.

In **1**, intermolecular hydrogen bonds are formed between neighboring complex anions and water molecules of crystallization to construct two-dimensional sheet structures along the *bc* plane. The two-dimensional sheet structures in the *bc* plane are also formed in **1′** by the intermolecular hydrogen bonds between the neighboring complexes. These results are indicative of the strong ability of Amp^2−^ to induce hydrogen-bonding interactions.

#### 2.2.2. Crystal Structure of **2**

**Figure 4:**
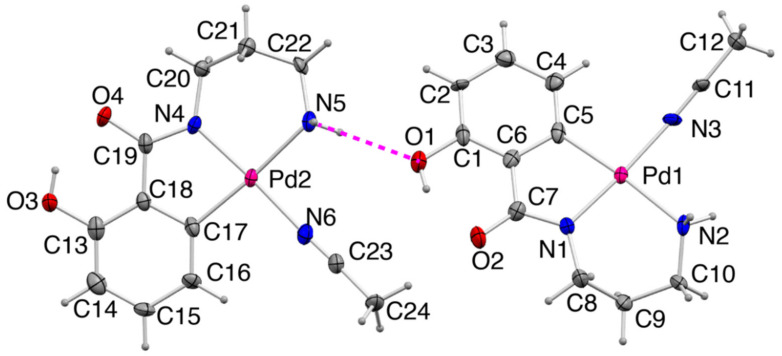
The asymmetric unit of **2** (50% probability levels). An intermolecular hydrogen bond is shown as magenta dashed line.

In the crystal of **2**, [Pd(Apr)(CH_3_CN)] also takes a general square planar coordination geometry (Figure 4 and Table 3). The asymmetric unit consists of two independent [Pd(Apr)(CH_3_CN)] molecules, and each molecule is stacked to form a centrosymmetric dimer structure by NH π interactions (the symmetric operation for Pd1 moiety is 1−x, 1−y, 2−z and for Pd2 moiety is 2−x, 2−y, 1−z). Despite the structural similarity with Amp^2−^, Apr^2−^ ligand coordinates to a Pd^II^ center with phenyl-*C*, amidato-*N*, and amino-*N* atoms. The difference in the coordination modes can be attributed to the combination of chelate rings. For a linear tridentate ligand in octahedral complexes, the combination of six-membered and five-membered chelates is more favored to afford *meridional* coordination geometry, whereas the combination of two six-membered chelates favors *facial* coordination geometry [15,16]. The coordination of a linear-tridentate ligand in square planar complexes may correspond to the coordination mode of the *meridional* type in the octahedral complexes. The observation of aromatic carbon coordination in the formation of complex **2** coincides with the larger stability gain by the combination of five- and six-membered chelates in the octahedral complexes. When Apr^2−^ ligand coordinates in the same way to Amp^2−^, the resulting formation of two six-membered rings corresponds to *facial* coordination in the octahedral complexes, but in square planar complexes, it is impossible. The average bond length between the Pd^II^ ion and the terminal amino-*N* atom, 2.13 Å, is significantly longer than those in **1** and **1′** (2.03 Å) because of a strong *trans* influence of phenyl-*C* donor [18]. The hydroxy group of the ligand forms an intramolecular hydrogen bond with the O atom of the carbonyl group.

### 2.3. Reaction of ***1*** with Nucleosides

**Figure 5:**
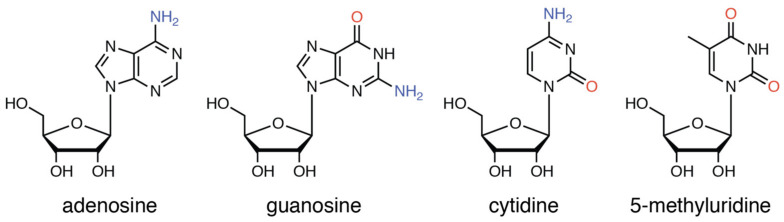
Chemical structures of the nucleosides employed in this study. Hydrogen-bond donors and acceptors are shown in blue and red color.

Although **1′** is suitable for the ligand exchange, KCl was easily contaminated as impurity during the synthesis. Thus, we employed **1** for the substitution study with nucleosides as the substitution reaction of the chloride ligand is readily proceeded in methanol. Reactivity and selectivity of [Pd(Amp)Cl]^−^ with four nucleosides, guanosine, adenosine, cytidine, and 5-methyluridine, were evaluated in methanol by ^1^H NMR spectroscopy (Figure 5). The reaction was performed by mixing **1** and the nucleoside in methanol.

#### 2.3.1. Reaction of **1** with Pyrimidine-Nucleosides

**Figure 6:**
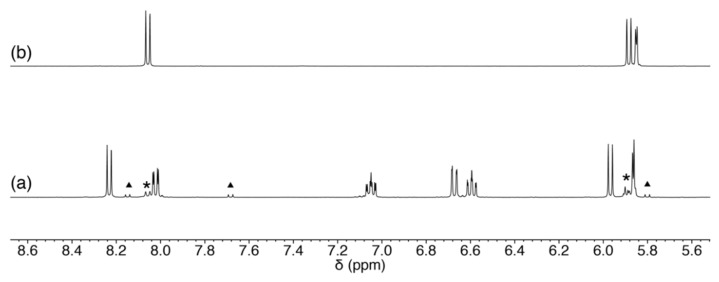
^1^H NMR spectra of (**a**) the reaction mixture of **1** and cytidine, (**b**) cytidine in CD_3_OD. The resonances from a minor product and unreacted cytidine are marked with triangles and asterisks, respectively.

**Figure 7:**
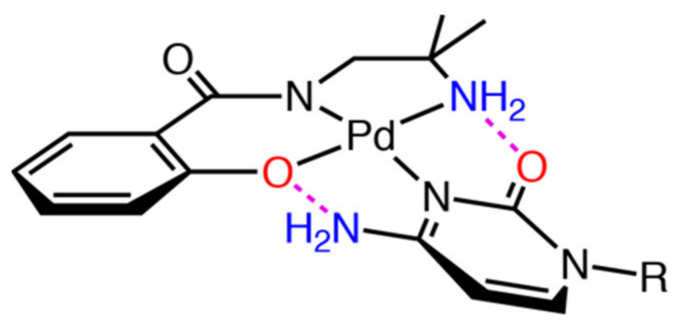
Chemical structure of **Pd-C** (R indicates the ribose moiety). Hydrogen-bond donor and acceptor are shown in blue and red, respectively. Dashed lines in magenta indicate intramolecular hydrogen bonds.

Two pyrimidine nucleosides, cytidine and 5-methyluridine, were evaluated. Cytidine was suitable for selective coordination to [Pd(Amp)] fragment because it has only one possible coordination site and both hydrogen-bond donor and acceptor sites were available within the proximity of the coordination site (2- and 4-position of the pyrimidine ring). The ^1^H NMR spectrum indicated the formation of [Pd(Amp)(cytidine)]-adduct (**Pd-C**) as shown in Figure 6. The broad resonances observed in the spectrum of **1** became converged in clear multiplets in the presence of cytidine, implying that **Pd-C** is reasonably stable without further ligand exchange reaction. The splitting and geminal coupling of the signal corresponding to the coordinating amino group implies the presence of nonequivalent protons by the intramolecular hydrogen bond (4.6–4.3 ppm in Figure S4, Supplementary Materials). In the spectrum, only a trace amount of minor product was observed (<5%), which was attributed to a negligible coordination mode such as carbonyl-*O* coordination. The observation of a selective formation of **Pd-C** indicated that it has a specific binding mode by the coordination of N-donor site on pyrimidine that was supported by two pairs of donor-acceptor interactions (Figure 7). This three-point interaction is reminiscent of the Watson–Crick base pairing in DNA.

**Figure 8:**
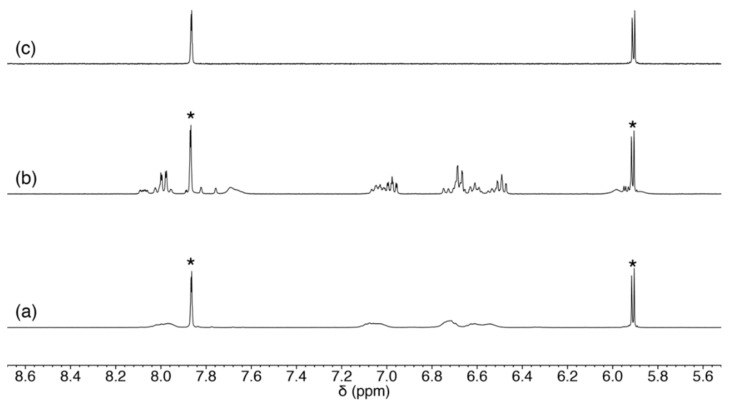
^1^H NMR spectra of (**a**) the reaction mixture of **1** and 5-methyluridine, (**b**) **1**, 5-methyluridine, and triethylamine, and (**c**) 5-methyluridine in CD_3_OD. The resonances for unreacted 5-methyluridine are marked with asterisks.

In the case of 5-methyluridine, on the other hand, no shift was observed for the resonances of both the nucleoside and [Pd(Amp)Cl]^−^, indicating that 5-methyluridine was not able to coordinate to the Pd center (Figure 8). In the presence of 1 equiv. of triethylamine, a few sets of resonances corresponding to 5-methyluridine, including unreacted, were observed. From the integration ratio, over 80% of 5-methyluridine remained unreacted, although the formation of a minor amount of [Pd(Amp)(5-methyluridine)]-adduct was observed. The resonances corresponding to [Pd(Amp)]-fragment became sharp and the formation of a new species [Pd(Amp)(OCD_3_)]^−^ was suggested. These results showed that two hydrogen-bond acceptors in 5-methyluridine distract its coordination to the [Pd(Amp)]-fragment due to the electrostatic repulsive force between O atoms of Amp^−^ ligand and 5-methyluridine. Thus, [Pd(Amp)]-fragment exhibited high selectivity on the coordination of pyrimidine-nucleosides, owing to the preference of hydrogen-bonding interactions between Amp^−^ and the pyrimidine moieties.

#### 2.3.2. Reaction of **1** with Purine-Nucleosides

**Figure 9:**
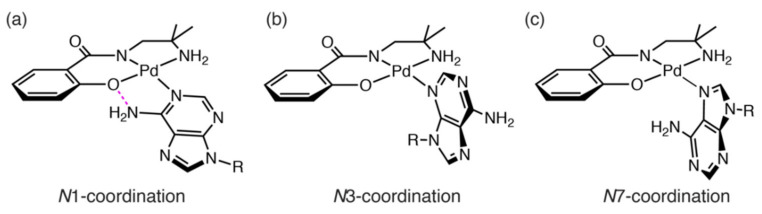
Three possible coordination modes for **Pd-A** (R indicates the ribose moiety). (**a**) *N*1-coordination mode, (**b**) *N*3-coordination mode, and (**c**) *N*7-coordination mode. Hydrogen bonds are shown in magenta.

**Figure 10:**
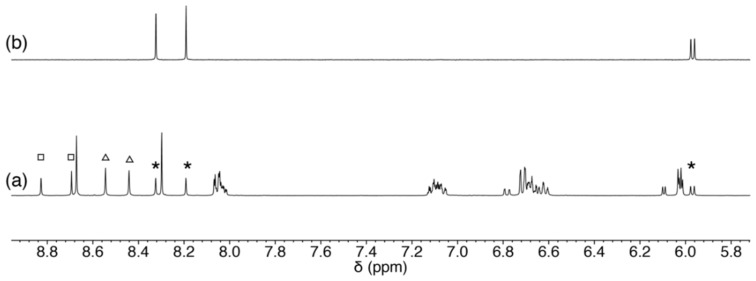
^1^H NMR spectra of (**a**) the reaction mixture of **1** and adenosine, and (**b**) adenosine in CD_3_OD. The resonances of aryl-H atoms for two minor products and adenosine are marked in the spectrum (**Pd-A2**: open triangle; **Pd-A3**: open square; adenosine: asterisk).

Two purine-nucleosides, adenosine and guanosine, were evaluated as well as the pyrimidine-nucleosides. Due to the increasing possible coordination sites (*N*1-, *N*3-, and *N*7-position), purine-nucleosides might afford different products compared with the previous cases. Adenosine possesses a hydrogen-bond donor at 6-position and no hydrogen-bond acceptor exists at the coordination site. For [Pd(Amp)(adenosine)]-adduct (**Pd-A**), three different coordination modes are possible depending on which N atom is coordinated (Figure 9). The ^1^H NMR spectrum indicated that the product is a mixture of **Pd-A** (Figure 10). The resonances according to the protons at adenine moiety imply that three species (**Pd-A1**, **Pd-A2**, and **Pd-A3**) are present in the reaction solution. The formation ratio of the products was determined to be **Pd-A1**:**Pd-A2**:**Pd-A3** = 6:3:2 from the integration of the resonances of the aryl-H of the adenine moiety. The *N*1-coordination mode is favorable because it is supported by a hydrogen bond (Figure 9a). Such intramolecular hydrogen-bond formation with adenine was reported in a Pd^II^ complex with coordination at the *N*9 position of adenine, although the *N*9 position is not available for coordination in our current system [19]. In contrast, no intramolecular hydrogen-bond interactions are expected for the other two modes (Figure 9b,c). Moreover, the *N*3 coordination must endure a large degree of steric hindrance between the ligand and ribose moiety. Thus, the major product **Pd-A1** is in the *N*1-coordination mode, and the two other sets of signals **Pd-A2** and **Pd-A3** correspond to the *N*7- and *N*3-coordination modes, respectively.

**Figure 11:**
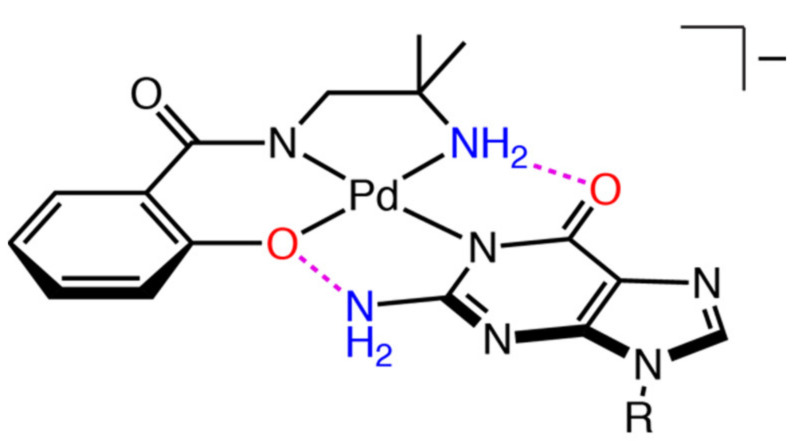
Chemical structure of **Pd-G** anion (R indicates the ribose moiety). Hydrogen-bond donor and acceptor are shown in blue and red, respectively. Dashed lines in magenta indicate intramolecular hydrogen bonds.

**Figure 12:**
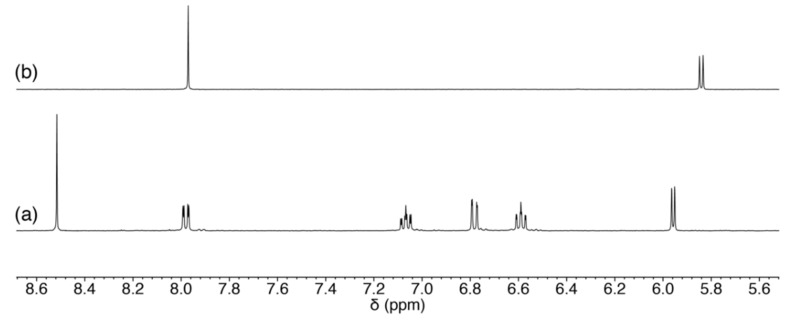
^1^H NMR spectra of (**a**) the reaction mixture of **1** and guanosine, and (**b**) guanosine in CD_3_OD.

Guanosine is structurally similar to cytidine in terms of the position of hydrogen-bond donors and acceptors (2- and 6-position, respectively). Although guanosine need to be deprotonated for the coordination at the *N*1 atom, formation of double intermolecular hydrogen bonds is expected for guanosine (Figure 11). The ^1^H NMR spectrum of 1:1 mixture of **1** and guanosine in CD_3_OD exhibited a sole set of resonances corresponding to [Pd(Amp)(guanosine)]-adduct (**Pd-G**), as shown in Figure 12. As observed in the case of cytidine, geminal coupling of the signals corresponding to the coordinating amino group was also observed, indicating *N*1 coordination of guanine moiety (5.3–5.0 ppm in Figure S7). It is to be noted that *N*7 is the most nucleophilic position in a neutral guanine moiety, and as a result, it is the primary binding site [20]. The *N*1 coordination in **Pd-G** without addition of base is indicative of the strong preference toward formation of intramolecular hydrogen bonds.

#### 2.3.3. Scrambling Reaction of **1** with Two Nucleosides

**Figure 13:**
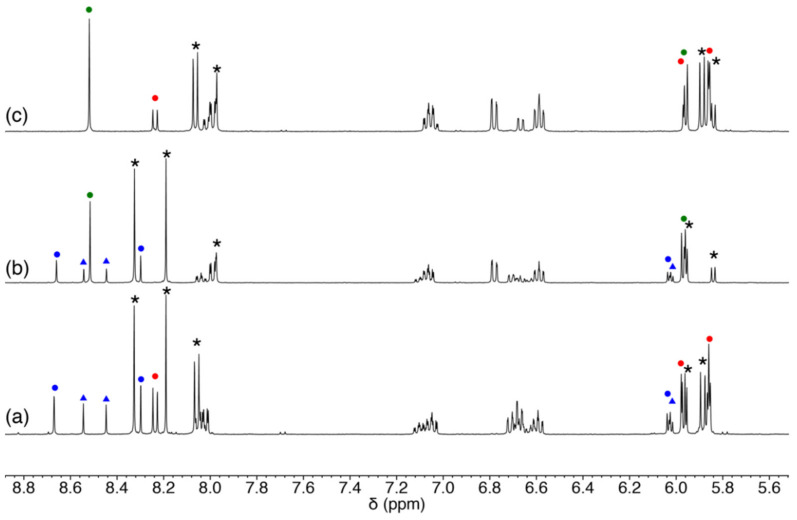
^1^H NMR spectra of **1** with two different nucleosides in CD_3_OD. (**a**) Adenosine and cytidine, (**b**) adenosine and guanosine, and (**c**) guanosine and cytidine. The resonances attributed to the major and minor products for each nucleoside are indicated with solid circles and triangles, respectively (the resonances for Amp^2−^ ligand for each species are not marked). **Pd-A**, **Pd-G**, and **Pd-C** species are shown in blue, green, and red, respectively. Asterisks indicate unreacted nucleoside.

To investigate the selectivity among nucleosides, complex **1** was reacted with two kinds of nucleosides at the same time. The Pd complex **1**, nucleoside-1, and nucleoside-2 were reacted in methanol at room temperature in a 1:1:1 molar ratio. 5-Methyluridine was excluded because it did not react with **1**. All sets of the combinations from two kinds of nucleosides were tested: adenosine vs. cytidine, adenosine vs. guanosine, and guanosine vs. cytidine (Figure 13).

In all cases, a mixture of a few Pd-nucleoside adducts was obtained. In the presence of adenosine and cytidine, **Pd-A1**, **Pd-A2**, and **Pd-C** were formed and formation of **Pd-A3** was negligible. From the formation ratio of **Pd-A1**:**Pd-A2**:**Pd-C** = 3:2:6, the coordination of cytidine is significantly more favorable than adenosine because of the formation of the double intramolecular hydrogen bonds. In the case of adenosine and guanosine, both **Pd-A1** and **Pd-A2** were observed, similar to the previous case. The formation ratio of **Pd-A1**:**Pd-A2**:**Pd-G** = 3:2:10 indicated that the coordination of guanosine is about twice as favorable as that of adenosine. These two experiments are indicative of the stabilization effect of double intramolecular hydrogen-bond formation on the coordination.

Finally, the coordination ability of guanosine and cytidine was compared. The NMR spectrum showed only two species, **Pd-G** and **Pd-C**, in the reaction. The formation ratio was found to be **Pd-G**:**Pd-C** = 2:1, which is reasonably consistent with the ratio expected from the previous two experiments, (**Pd–G**/**Pd-A)**:(**Pd-C**/**Pd-A)** = 10:6. Thus, guanosine has the highest preference among the four nucleosides. Considering the number of hydrogen-bonding interactions of guanosine and cytidine with [Pd(Amp)]-moiety, the formation ratio should be comparable. As the anionic *N*1 position of the guanine moiety is more nucleophilic, however, the coordination of guanosine was more favorable than that of cytidine.

## 3. Materials and Methods

### 3.1. Measurements

Elemental analyses (C, H, and N) were performed at the Research Institute for Instrumental Analysis, Kanazawa University. ^1^H NMR measurements were carried out at 22 °C on a JEOL 400SS spectrometer. Chemical shifts were referenced to the solvent residual peak [21].

### 3.2. Materials

All the chemicals were used as received without further purification. The ligand precursors *N*-(2-amino-2-methylpropyl)salicylamide (H_2_Amp), *N*-3-aminopropylsalicylamide (H_2_Apr), and the starting material of Pd complex, [PdCl_2_(CH_3_CN)_2_], were synthesized according to the previously reported procedure [15,17]. 

### 3.3. Preparations

N(CH_3_)_4_[Pd(Amp)Cl]·H_2_O (**1**). To an acetonitrile solution (20 mL) of [PdCl_2_(CH_3_CN)_2_] (51.4 mg, 0.20 mmol) was added a solid H_2_Amp (41.5 mg, 0.20 mmol) and triethylamine (28 µL). After stirring the mixture for 10 min, a solid (CH_3_)_4_NOH·5H_2_O (36.2 mg, 0.20 mmol) was added followed by stirring at room temperature overnight. The reaction solution was concentrated to 2 mL by Ar gas bubbling. Diethyl ether solution was diffused to the solution to give orange, needle-shaped crystals. Yield: 65.8 mg, 53%. Anal. Calcd for N(CH_3_)_4_[Pd(Amp)Cl]·H_2_O = C_15_H_28_ClN_3_O_3_Pd: C, 40.92; H, 6.41; N, 9.54%. Found: C, 40.64; H, 6.25; N, 9.54%. ^1^H NMR (399 MHz, CD_3_OD): δ 7.97 (broad, 1H, aryl-H), 7.05 (broad, 1H, aryl-H), 6.72 (broad, 1H, aryl-H), 6.58 (broad, 1H, aryl-H), 4.62 (s, 2H, NH_2_), 3.41 (s, 2H, CH_2_), 3.19 (s, 12H, CH_3_), 1.40 (s, 6H, CH_3_).

[Pd(Amp)(CH_3_CN)]·CH_3_CN (**1′**). To an acetonitrile solution (10 mL) of [PdCl_2_(CH_3_CN)_2_] (25.9 mg, 0.10 mmol) was added a solid H_2_Amp (20.8 mg, 0.1 mmol). After dissolving the ligand completely, a methanolic solution (5 mL) of KO*^t^*Bu (22.3 mg, 0.20 mol) was added to the mixture, followed by stirring overnight. The reaction solution was concentrated to 3 mL by Ar gas bubbling. The residue was filtered, and the filtrate was crystallized by slow evaporation of the solvent. Yield: 22.4 mg, 57%. Anal. Calcd for [Pd(Amp)(CH_3_CN)]·1.3H_2_O = C_13_H_19.6_N_3_O_3.3_Pd: C, 41.40; H, 5.24; N, 11.14%. Found: C, 41.85; H, 5.49; N, 10.60%. ^1^H NMR (399 MHz, CD_3_OD): δ 8.00 (m, 1H, aryl-H), 7.06 (dd, *J* = 8.6, 6.8 Hz, 1H), 6.71 (d, *J* = 8.2 Hz, 1H), 6.58 (dd, *J* = 8.3, 6.8 Hz, 1H), 3.82 (s, 2H, CH_2_), 2.52 (s, 3H, CH_3_), 1.43 (d, *J* = 7.6 Hz, 6H).

[Pd(Apr)(CH_3_CN)] (**2**). To an acetonitrile solution (8 mL) of [PdCl_2_(CH_3_CN)_2_] (25.6 mg, 0.10 mmol) was added a solid H_2_Apr (19.4 mg, 0.1 mmol) and triethylamine (14 µL). After dissolving the ligand completely, a methanolic solution (3 mL) of KO*^t^*Bu (11.0 mg, 0.10 mol) was added to the mixture, followed by stirring overnight. The reaction solution was filtered to remove white precipitate. The filtrate was evaporated to dryness and dissolved in acetonitrile (2 mL). Diethyl ether vapor was diffused to the solution to give yellow microcrystals. Yield: 7.3 mg, 23%. This compound was extremely hygroscopic and unable to perform elemental analysis. ^1^H NMR (399 MHz, CD_3_CN): δ 6.76 (m, 1H, aryl-H), 6.59 (dd, *J* = 7.4, 0.7 Hz, 1H, aryl-H), 6.33 (dd, *J* = 8.2, 0.9 Hz, 1H, aryl-H), 3.22 (m, 2H, CH_2_), 2.66 (m, 4H, CH_2_, and NH_2_), 1.57 (m, 2H, CH_2_).

### 3.4. Crystallography

Crystallographic data are summarized in Table 4. Single-crystal X-ray diffraction data were obtained with a Rigaku XtaLAB AFC11 diffractometer with graphite-monochromated Mo Kα radiation (λ = 0.71073 Å). A single crystal was mounted with a glass capillary and flash-cooled with a cold N_2_ gas stream. Data were processed using the CrysAlisPro software packages. The structure was solved by intrinsic phasing methods using the SHELXT [22] software packages and refined on *F*^2^ (with all independent reflections) using the SHELXL [23] software packages. The non-hydrogen atoms were refined anisotropically. The hydrogen atoms except for OH and CH_3_ groups were located at the calculated positions and refined isotopically using the riding models. The hydrogen atoms for OH and CH_3_ groups were located by difference Fourier maps and allowed to rotate. The O–H atoms for water molecules were located at the position suitable for hydrogen bonds and refined freely with a restrained distance. In the case of **2**, only tiny single-crystals were obtained. As it is difficult to apply a sufficient absorption correction to a tiny crystal based on the actual size, the *R_int_* value for **2** is apparently large. However, the quality of the crystal itself was reasonable. The anisotropic refinement for the acetonitrile ligands was restrained to obtain reasonable parameters. The Cambridge Crystallographic Data Centre (CCDC) deposition numbers are included in Table 4.

### 3.5. Reaction of ***1*** with Nucleosides

The reactions were performed by mixing a methanol solution of **1** with a solid nucleoside. Although some nucleosides are hardly soluble to typical organic solvents such as methanol and dimethylformamide, they dissolved well in methanol in the presence of **1**. A typical procedure for reactions of **1** with nucleosides is as follows. 

A 5 mL methanolic solution of **1** (20 µmol) was added to a solid nucleoside (20 µmol) followed by stirring overnight. Half of the reaction mixture was taken and evaporated to dryness by Ar gas bubbling. The resulting yellow residue was dried in vacuo for 1 h. The residue was dissolved in ca. 0.7 mL of CD_3_OD, followed by ^1^H NMR measurement. 

## 4. Conclusions

Palladium(II) complexes containing unsymmetric tridentate ligand, Amp^2−^, and Apr^2−^, were synthesized and crystallographically characterized. Although Amp^2−^ exhibited a typical O–N–N coordination mode, Apr^2−^ formed a Pd–C bond, resulting an observation of the C–N–N coordination mode. The Pd complex with Amp^2−^, **1**, was employed for evaluating the effect of stereoselective intramolecular hydrogen-bonding interaction of four nucleosides (adenosine, guanosine, cytidine, and 5-methyluridine). Three of the four nucleosides resulted in coordination to the [Pd(Amp)]-fragment. In the case of 5-methyluridine, in which no intramolecular hydrogen bond is expected upon coordination, [Pd(Amp)(5-methyluridine)]-adduct was not formed. By contrast, the other three nucleosides, which form intramolecular hydrogen bonds upon coordination, were coordinated to the [Pd(Amp)]-fragment. These results clearly show that hydrogen-bonding interactions play an important role upon coordination to the [Pd(Amp)]-fragment. Preference of the nucleosides was further evaluated by reacting **1** with two different nucleosides, and guanosine showed the highest selectivity among the series.

## Figures and Tables

**Table 1:** Selected bond parameters for **1**.

Atom–Atom	Length/Å	Atom–Atom–Atom	Angle/°
Pd(1)–O(1)	1.9877 (13)	N(1)–Pd(1)–O(1)	93.70 (6)
Pd(1)–N(1)	1.9647 (14)	N(1)–Pd(1)–N(2)	83.87 (6)
Pd(1)–N(2)	2.0264 (16)	O(1)–Pd(1)–N(2)	177.15 (5)
Pd(1)–Cl(1)	2.3389 (4)	N(1)–Pd(1)–Cl(1)	176.50 (4)
		O(1)–Pd(1)–Cl(1)	89.79 (4)
		N(2)–Pd(1)–Cl(1)	92.64 (4)

**Table 2:** Selected bond parameters for **1′**.

Atom–Atom	Length/Å	Atom–Atom–Atom	Angle/°
Pd(1)–O(1)	1.9771 (19)	N(1)–Pd(1)–O(1)	94.73 (8)
Pd(1)–N(1)	1.956 (2)	N(1)–Pd(1)–N(2)	82.77 (9)
Pd(1)–N(2)	2.025 (2)	O(1)–Pd(1)–N(2)	176.79 (8)
Pd(1)–N(3)	2.027 (2)	N(1)–Pd(1)–N(3)	176.99 (8)
		O(1)–Pd(1)–N(3)	88.02 (8)
		N(2)–Pd(1)–N(3)	94.44 (9)

**Table 3:** Selected bond parameters for **2**.

Atom–Atom	Length/Å	Atom–Atom–Atom	Angle/°
Pd(1)–C(5)	1.982 (5)	N(1)–Pd(1)–C(5)	81.8 (2)
Pd(1)–N(1)	1.974 (5)	N(1)–Pd(1)–N(2)	92.2 (2)
Pd(1)–N(2)	2.144 (4)	C(5)–Pd(1)–N(2)	173.2 (3)
Pd(1)–N(3)	1.986 (5)	N(1)–Pd(1)–N(3)	175.65 (17)
Pd(2)–C(17)	1.981 (6)	C(5)–Pd(1)–N(3)	95.1 (2)
Pd(1)–N(4)	1.998 (4)	N(2)–Pd(1)–N(3)	90.72 (19)
Pd(1)–N(5)	2.121 (5)	N(4)–Pd(2)–C(17)	81.8 (2)
Pd(1)–N(6)	2.013 (5)	N(4)–Pd(2)–N(5)	94.33 (18)
		C(17)–Pd(2)–N(5)	173.84 (19)
		N(4)–Pd(2)–N(6)	176.3 (2)
		C(17)–Pd(2)–N(6)	95.3 (2)
		N(5)–Pd(2)–N(6)	88.41 (19)

**Table 4:** Crystallographic data and refinement parameters of **1′** and **2**.

Complex	1	1′	2
Empirical formula	C_15_H_28_ClN_3_O_3_Pd	C_15_H_20_N_4_O_2_Pd	C_12_H_15_N_3_O_2_Pd
Formula weight	440.25	394.75	339.67
Crystal system	Monoclinic	Monoclinic	Triclinic
Crystal dimensions/mm	0.19 × 0.12 × 0.06	0.21 × 0.15 × 0.09	0.11 × 0.07 × 0.04
Space group	*P*2_1_/*c*	*P*2_1_/*c*	*P*−1
*a*/Å	11.8363 (3)	13.3692 (5)	8.7885 (4)
*b*/Å	8.7436 (2)	10.9488 (4)	10.8746 (5)
*c*/Å	18.3451 (5)	11.1827 (4)	13.2005 (5)
α/°			96.688 (4)
β/°	104.732 (3)	94.084 (3)	90.840 (3)
γ/°			104.073 (4)
*V*/Å^3^	1836.15 (8)	1632.73 (10)	1214.19 (9)
*Z*	4	4	4
*T*/K	100 (2)	100 (2)	100 (2)
*ρ*_calcd_/g·cm^−3^	1.593	1.606	1.858
*µ*/mm^−1^	1.173	1.149	1.526
*F*(000)	904	800	680
2*θ*_max_/◦	55	55	55
No. of reflections measured	17,469	11,616	17,415
No. of independent reflections	4207 (*R_int_* = 0.0244)	3737 (*R_int_* = 0.0390)	5543 (*R_int_* = 0.1488)
Data/restraints/parameters	4207/2/222	3737/0/203	5543/18/329
*R*_1_ ^1^ [*I* > 2.00 σ(*I*)]	0.0217	0.0320	0.0555
*wR*_2_ ^2^ (all reflections)	0.0558	0.0803	0.1348
Goodness of fit indicator	1.076	1.088	0.906
Highest peak, deepest hole/e Å^−3^	0.445, −0.439	0.627, −1.156	1.646, −1.379
CCDC deposition number	2153005	2153006	2153007

^1^*R*_1_ = Σ||Fo| − |Fc||/Σ|Fo|, ^2^ *wR*_2_ = [Σ(*w*(Fo^2^ − Fc^2^)^2^)/Σ*w*(Fo^2^)^2^]^1/2^.

## Data Availability

The crystallographic data are available from the Cambridge Crystallographic Data Centre (CCDC). Other data not presented in Supplementary Materials are available on request from the corresponding author.

## Supplementary Materials

The following supporting information can be downloaded at https://www.mdpi.com/article/10.3390/molecules27072098/s1. Table S1: Hydrogen-bonding distances and angles; Figure S1: ^1^H NMR spectrum of **1** in CD_3_OD; Figure S2: ^1^H NMR spectrum of **1′** in CD_3_OD; Figure S3: ^1^H NMR spectrum of **2** in CD_3_CN; Figure S4: ^1^H NMR spectra of (a) reaction mixture of **1** and cytidine, (b) cytidine in CD_3_OD; Figure S5: ^1^H NMR spectra of (a) reaction mixture of **1** and 5-methyluridine, (b) **1**, 5-methyluridine, and triethylamine, and (c) 5-methyluridine; cytidine in CD_3_OD; Figure S6: ^1^H NMR spectra of (a) reaction mixture of **1** and adenosine, (b) adenosine in CD_3_OD; Figure S7: ^1^H NMR spectra of (a) reaction mixture of **1** and guanosine, (b) guanosine in CD_3_OD.
