# Supplementary material for: Selective Formation of Intramolecular Hydrogen-Bonding Palladium(II) Complexes with Nucleosides Using Unsymmetrical Tridentate Ligands
Source: Molecules. 2022 Mar 24;27(7):2098. doi: 10.3390/molecules27072098 (PMC9000330; doi:10.3390/molecules27072098)
Supplement: Supplementary file 1 [file molecules-27-02098-s001.zip › molecules-1633525-supplementary.pdf]

Supporting Information

Selective Formation of Intermolecular Hydrogen-bonding Palladium(II) Complexes with Nucleosides Using Unsymmetrical Tridentate Ligands

Ryoji Mitsuhashi,[1,*] Yuya Imai,[2] Takayoshi Suzuki,[3] Yoshihito Hayashi[2]

[1] Institute of Liberal Arts and Science, Kanazawa University; Kakuma, Kanazawa, Ishikawa 920-1192, Japan
[2] Department of Chemistry, Kanazawa University; Kakuma, Kanazawa, Ishikawa 920-1192, Japan
[3] Institute for Interdisciplinary Science, Okayama University,3-1-1 Tsushima-naka, Okayama, 700-8530, Japan
E-mail mitsuhashi@staff.kanazawa-u.ac.jp

Contents:

1. X-Ray crystallography

Table S1. Hydrogen-bond distances and angles.

2.^{1}H NMR Spectroscopy

Figure S1. ^{1}H NMR spectra of (a) reaction mixture of **1** in CD_3OD.
Figure S2. ^{1}H NMR spectra of (a) reaction mixture of **1’** in CD_3OD.
Figure S3. ^{1}H NMR spectra of (a) reaction mixture of **2** in CD_3CN.
Figure S4. ^{1}H NMR spectra of (a) reaction mixture of **1** and cytidine, and (b) cytidine in CD_3OD.
Figure S5. ^{1}H NMR spectra of (a) reaction mixture of **1** and 5-methyluridine, (b) **1**, 5-methyluridine and triethylamine, and (c) 5-methyluridine in CD_3OD.
Figure S6. ^{1}H NMR spectra of (a) reaction mixture of **1** and adenosine, (b) adenosine in CD_3OD.
Figure S7. ^{1}H NMR spectra of (a) reaction mixture of **1** and guanosine, (b) guanosine in CD_3OD.

1. X-Ray crystallography

Table S1. Hydrogen-bond distances and angles.

	D–H···A			D–H / Å	H···A / Å	D···A /Å	D–H···A / °
**1**	N2	H2A	O2^{i}	0.91	1.95	2.8609(18)	177.1
	O3	H3A	O1	0.975(19)	1.82(2)	2.785(2)	169(4)
**1’**	N2	H2A	O2^{ii}	0.91	2.07	2.969(3)	171.9
	N2	H2B	O2^{iii}	0.91	2.06	2.904(3)	153.3
**2**	O1	H1	O2	0.84	1.87	2.608(6)	146.7
	N2	H2A	O3^{iv}	0.91	2.24	3.078(7)	153.1
	O3	H3A	O4	0.84	1.86	2.595(6)	146.0
	N5	H5A	O1	0.91	2.18	2.974(5)	145.3

Symmetry code: (i = ‘–x+1, y–1/2, –z+3/2’, ii = ‘x, –y+1/2, z+1/2’, iii = ‘–x+1, y+1/2,– z+1/2’, iv = x–1, y–1, z+1).

2.^{1}H NMR Spectroscopy

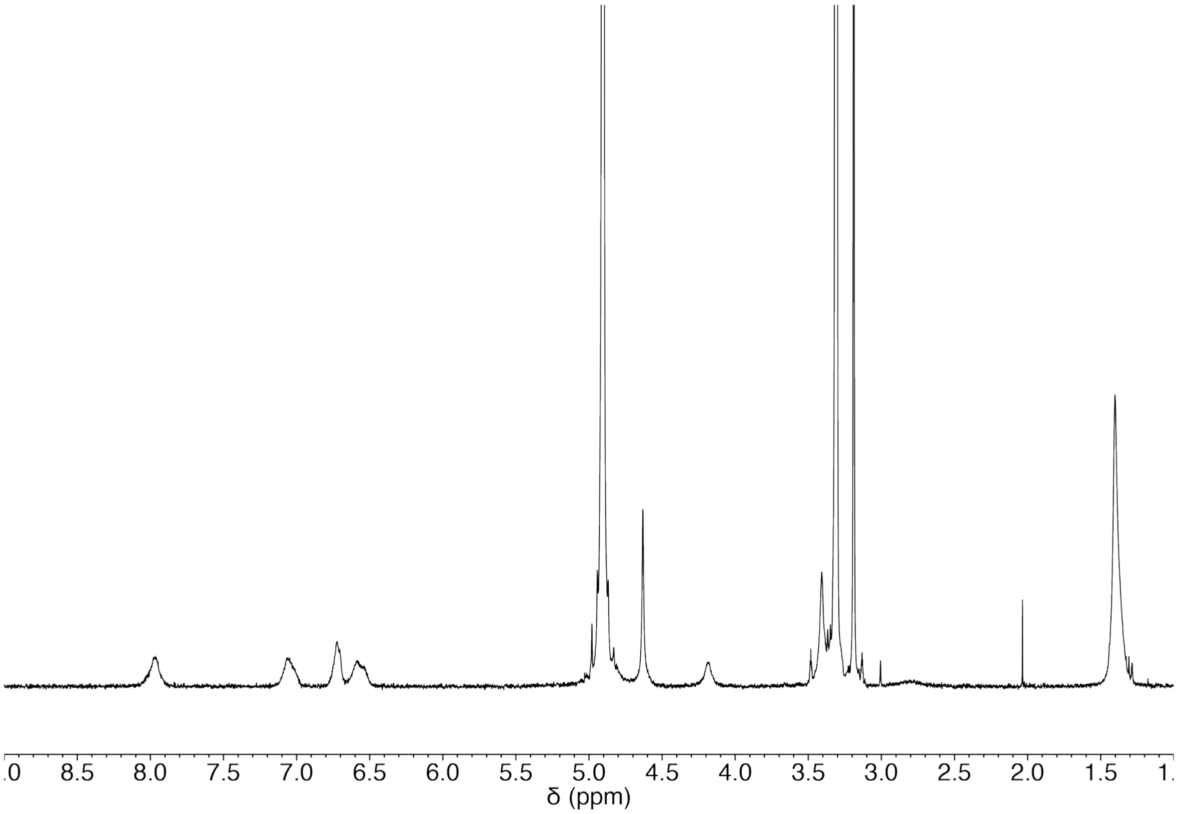

Figure S1. ^{1}H NMR spectra of (a) reaction mixture of **1** in CD_3OD.

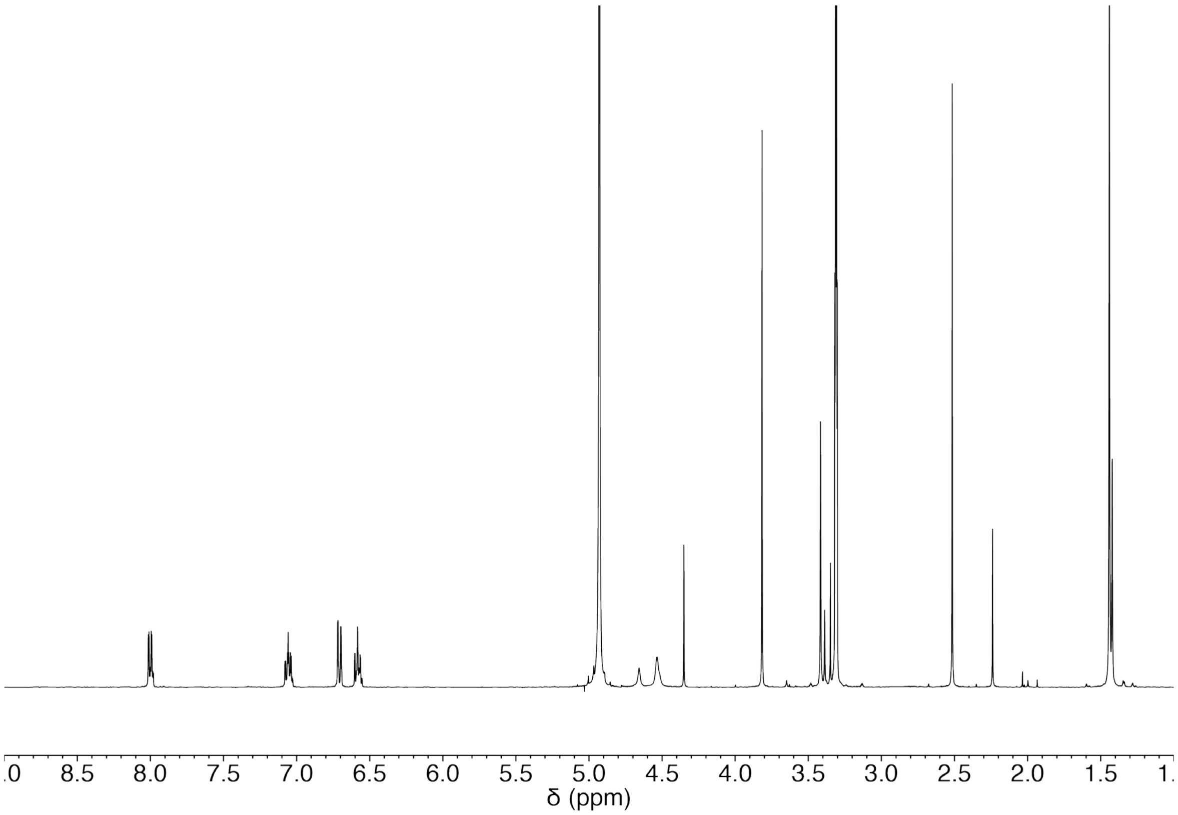

Figure S2. ^{1}H NMR spectra of (a) reaction mixture of **1’** in CD_3OD.

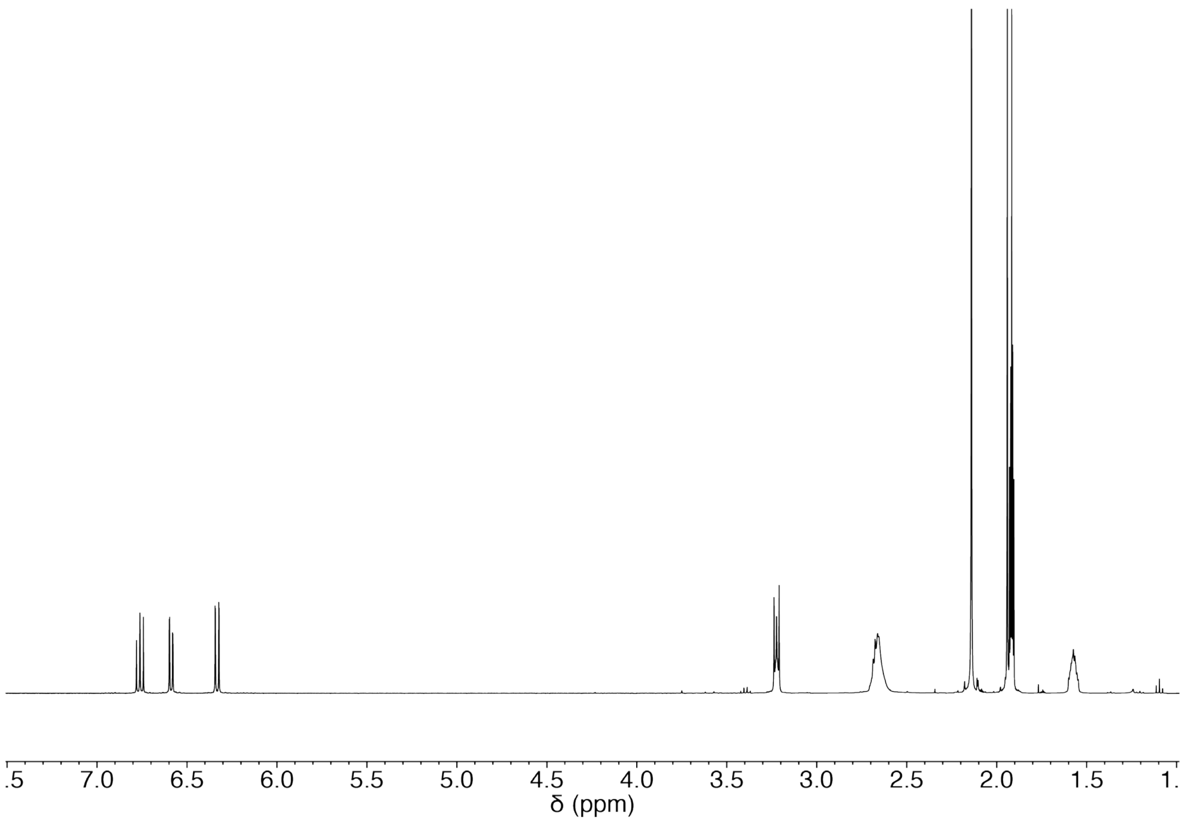

Figure S3. ^{1}H NMR spectra of (a) reaction mixture of **2** in CD_3CN.

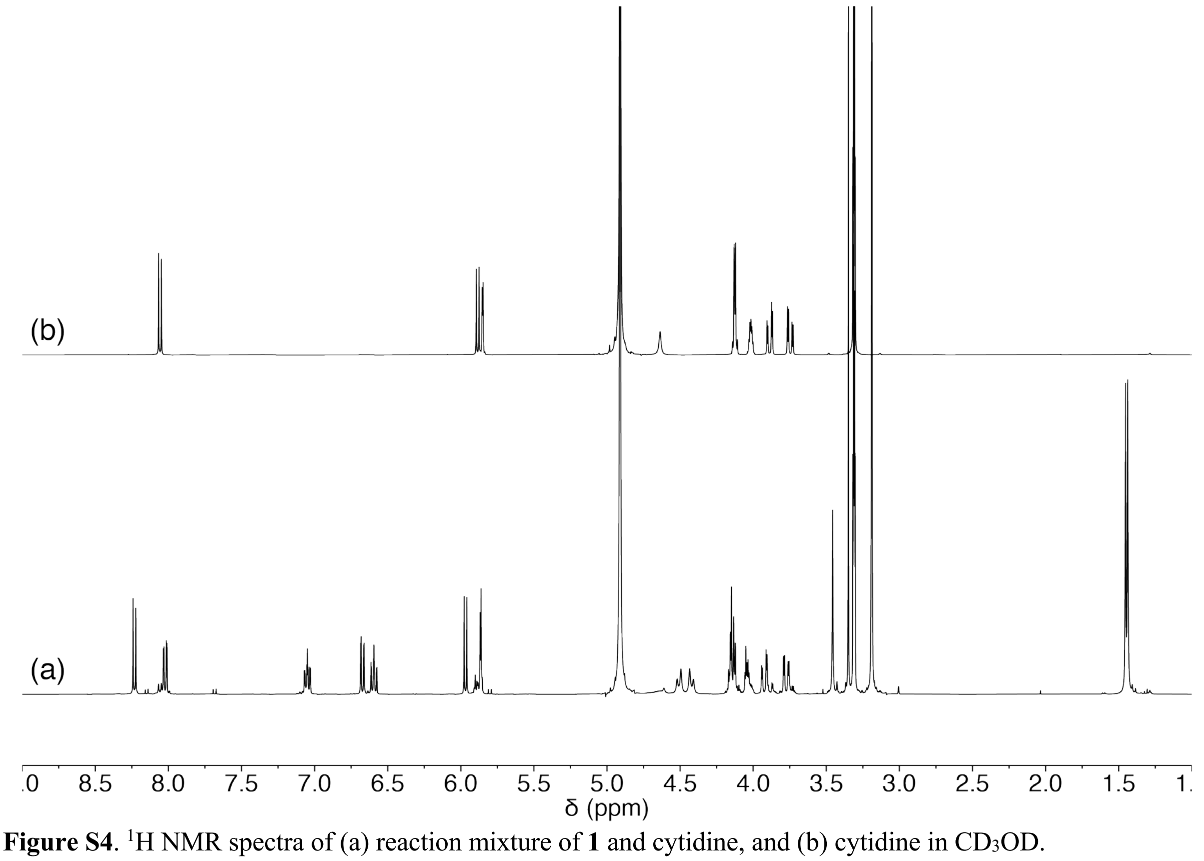

Figure S4. ^{1}H NMR spectra of (a) reaction mixture of **1** and cytidine, and (b) cytidine in CD_3OD.

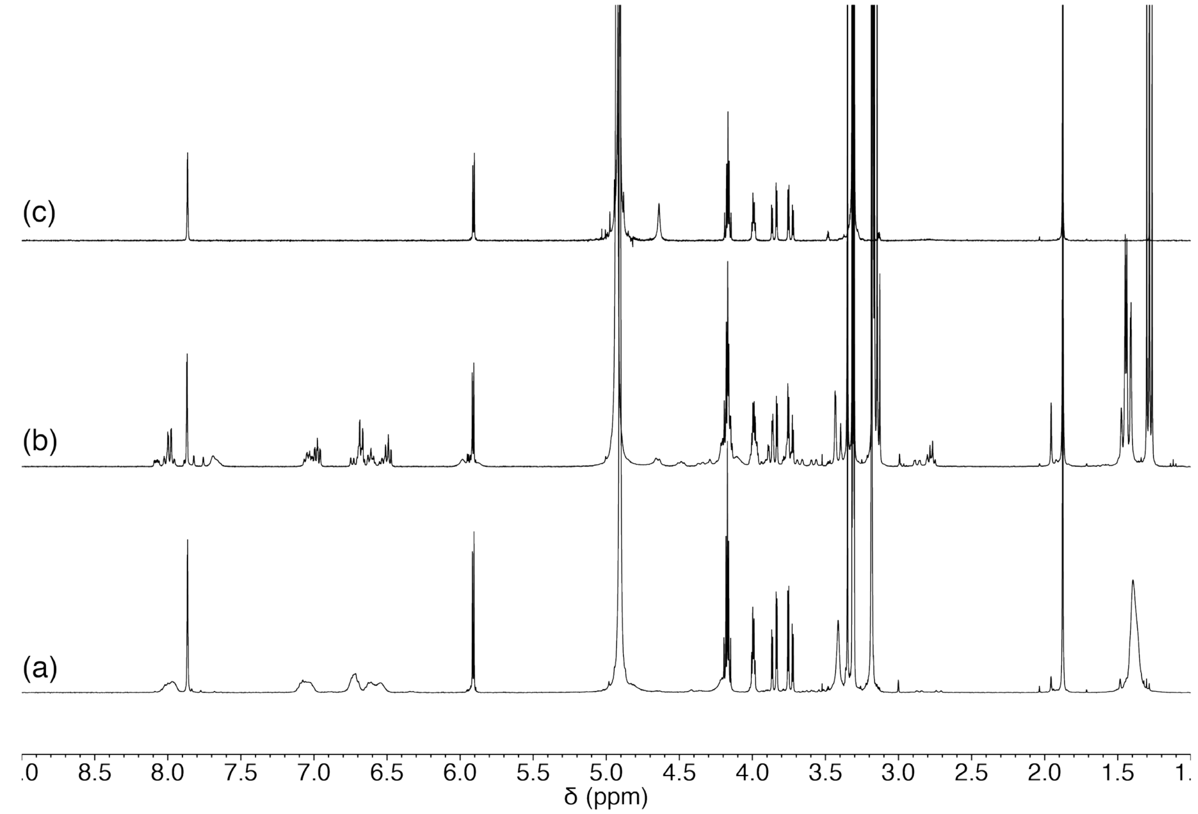

Figure S5. ^{1}H NMR spectra of (a) reaction mixture of **1** and 5-methyluridine, (b) **1**, 5-methyluridine and triethylamine, and (c) 5-methyluridine in CD_3OD.

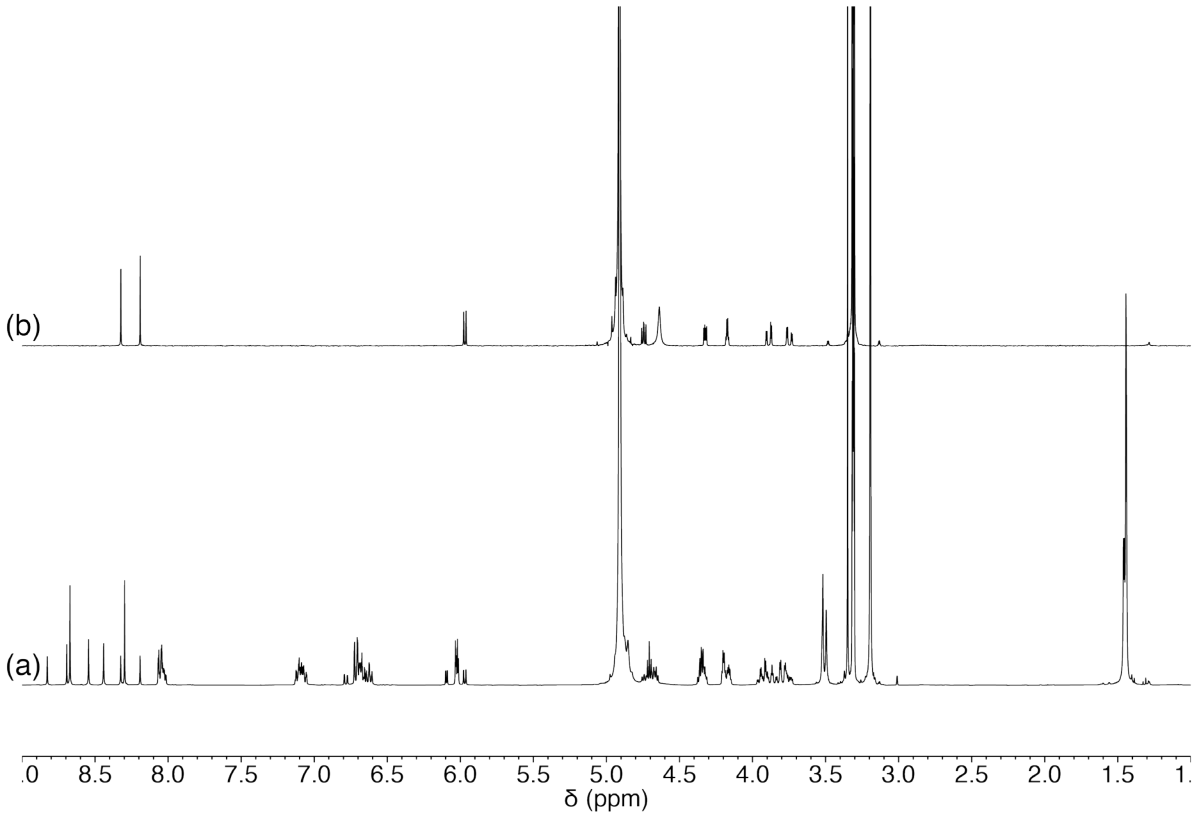

Figure S6. ^{1}H NMR spectra of (a) reaction mixture of **1** and adenosine, (b) adenosine in CD_3OD.

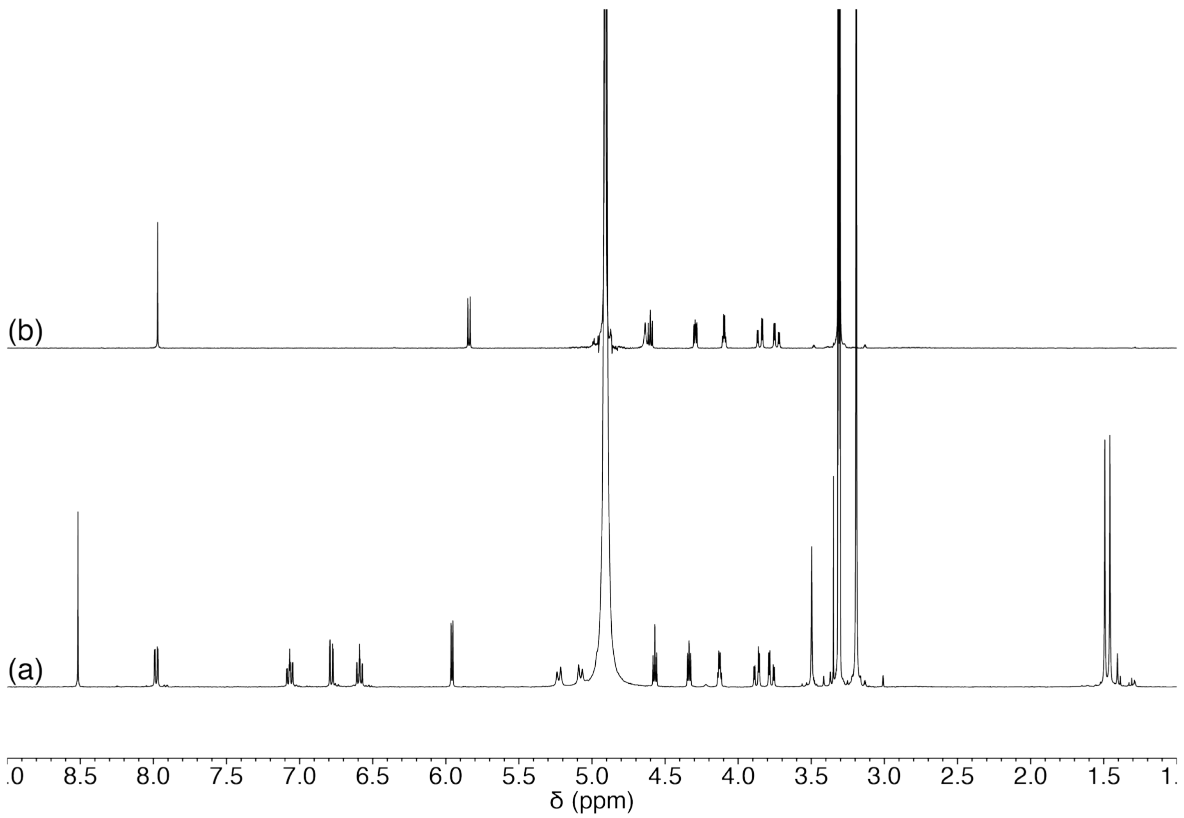

Figure S7. ^{1}H NMR spectra of (a) reaction mixture of **1** and guanosine, (b) guanosine in CD_3OD.
